# Digital Grading the Color Fastness to Rubbing of Fabrics Based on Spectral Reconstruction and BP Neural Network

**DOI:** 10.3390/jimaging9110251

**Published:** 2023-11-16

**Authors:** Jinxing Liang, Jing Zhou, Xinrong Hu, Hang Luo, Genyang Cao, Liu Liu, Kaida Xiao

**Affiliations:** 1 School of Computer Science and Artificial Intelligence, Wuhan Textile University, Wuhan 430200, China; jxliang@wtu.edu.cn (J.L.); 2115363094@mail.wtu.edu.cn (J.Z.); hxr@wtu.edu.cn (X.H.); 2 School of Automation, Qingdao University, Qingdao 266071, China; 3 School of Textile Science and Engineering, Wuhan Textile University, Wuhan 430200, China; 4 Analysis and Testing Center, Wuhan Textile University, Wuhan 430200, China; liuliuwtu@163.com; 5 School of Design, University of Leeds, Leeds LS2 9JT, UK; k.xiao1@leeds.ac.uk

**Keywords:** textile fabrics, color fastness, digital grading, spectral reconstruction, BP neural network

## Abstract

To digital grade the staining color fastness of fabrics after rubbing, an automatic grading method based on spectral reconstruction technology and BP neural network was proposed. Firstly, the modeling samples are prepared by rubbing the fabrics according to the ISO standard of 105-X12. Then, to comply with visual rating standards for color fastness, the modeling samples are professionally graded to obtain the visual rating result. After that, a digital camera is used to capture digital images of the modeling samples inside a closed and uniform lighting box, and the color data values of the modeling samples are obtained through spectral reconstruction technology. Finally, the color fastness prediction model for rubbing was constructed using the modeling samples data and BP neural network. The color fastness level of the testing samples was predicted using the prediction model, and the prediction results were compared with the existing color difference conversion method and gray scale difference method based on the five-fold cross-validation strategy. Experiments show that the prediction model of fabric color fastness can be better constructed using the BP neural network. The overall performance of the method is better than the color difference conversion method and the gray scale difference method. It can be seen that the digital rating method of fabric staining color fastness to rubbing based on spectral reconstruction and BP neural network has high consistency with the visual evaluation, which will help for the automatic color fastness grading.

## 1. Introduction

Color fastness is the expression of the color of a textile to various effects during the processing and use of the material, and it is a very important indicator when testing the color quality of fabrics [1,2,3]. Color fastness of textile fabrics is usually determined by assessing the discoloration of the sample, or by assessing the staining of the undyed lining fabric. Expressed as a number of grades, the color fastness is usually divided into five grades with a half grade between two adjacent grades, forming five grades and nine steps. Generally, the higher the grade, the better the color fastness, and, the lower the grade, the worse the color fastness.

For color fastness grading, the traditional method mainly uses the artificial visual method [4]. The color difference between the sample to be rated and the reference sample is usually visually observed by a specially trained professional in a dark room and under a standard light box. The visually observed color difference in textile samples is then compared with the standard grading gray card under the same observed conditions. The grade of the standard grading gray card closest to the visually observed color difference in textile fabrics is assigned as the color fastness grade of it. However, due to the subjective differences in the work experience and physiological state of the person, the artificial visual method will inevitably affect the final rating results. In addition, the artificial visual method can only help to estimate the approximate differences in tested samples based on human perception experience; it does not allow for quantitative analysis of visually perceived color differences. More importantly, the practical application of the artificial visual method is also inefficient.

With the development of technology, the grading of color fastness based on color measurement equipment such as spectrophotometers is used for fabric quality evaluation. As one of the precise optical measuring instruments, the spectrophotometer can accurately measure the spectral reflectance of the fabric, and the color data of the fabric can be calculated through colorimetric theory [5,6]. However, these kinds of instruments have their limitations when used for color fastness evaluation. Firstly, the color measurement results of the spectrophotometer just represent the average color of a circle area that is determined by the measuring aperture. Second, the spectrophotometers are not suitable for color measurement and evaluation of textile fabrics with complex and fine color patterns. In addition, the spectrophotometer can only complete one color measurement at a time; the measurement efficiency is low compared with the digital-imaging-based color measurement method, which can perform multiple points of color measurement through one capture and image processing techniques.

Recently, digital-imaging-based methods have been used for digital grading color fastness of fabric [7], where the color fastness is calculated based on the captured images of fabric and the mathematical grading method. In terms of the digital-imaging-based methods, the extracted RGB values are first converted to the CIEXYZ tristimulus and then converted to the CIELab values. Based on the calculated CIELab values, the color difference between the tested and reference fabric can be calculated [8,9]. Hence, the color fastness can be graded referring to the calculated color difference.

Two different types of digital color fastness grading methods have been proposed. The first type is called the color difference conversion method [10,11], and the second is called the gray scale difference method [12,13]. For the first type of method, using the calculated CIELab values from captured RGB values, the CIEDE2000 color difference is calculated between the tested and reference fabric, and the relationship between the CIEDE2000 color difference and standard color fastness grades are fitted by mathematical algorithms such as linear regression. With the established regression model, the color fastness of the newly tested fabric will be predicted [10,11]. For the gray scale difference method, the color image of standard grading gray card of color fastness captured with a digital camera is first converted to the grayscale image. Then, the grayscale difference between a pair of gray patches of each grade is calculated. After that, the grayscale differences are fitted with their corresponding color fastness grades using the gray scale difference method. With the fitted curve between the grayscale difference and corresponding grades, the newly tested fabric will be predicted for its color fastness based on the gray image difference between rubbed and unrubbed areas [12,13].

However, for the digital-imaging-based methods, due to the extracted RGB values being imaging-condition-related, if we do not perform color correction on the digital camera in advance, the calculated CIELab values are not the groundtruth color attributes of the tested fabric sample, which will inevitably contribute grading error to the grading results. In addition, even if the color correction has been performed on a digital camera, we can only acquire the color values under one specific illuminant, which will limit the using of the method if we should evaluate the color difference or color fastness grades under several different illuminants. In recent years, with the fast development of spectral reconstruction, it has been widely used in many fields as it can recover the ’fingerprint’ of the color values, the spectral reflectance. With the reconstructed spectral reflectance, the color values under any different illuminant are easily calculated based on the colorimetry. Furthermore, with the optimized spectral reconstruction algorithms published in recent years [14,15,16], the spectral reconstruction accuracy of the colorimetric has been obviously improved compared with the traditional color correction method, such as the classical polynomial-based color correction method [17]. Indeed, measuring fabric color using spectral reconstruction technology has been widely studied and achieved good results [18,19,20].

To further promote the application of spectral reconstruction technology in the textile industry, we propose the digital grading method for the color fastness to rubbing of textiles based on spectral reconstruction technology and BP (Backward Propagation) neural network modeling. Different from the existing methods that directly calculate the color data from RGB values, the spectral reconstruction technology can help us to acquire the spectral reflectance of the captured image of fabrics, which will provide the basis to calculate the groundtruth color data of the fabrics. In addition, the BP neural network is selected to model the relationship between the color attributes difference in the tested fabric and the corresponding color fastness grades. BP neural network has the advantages of learning ability and adaptability, and has been widely used in solving complex nonlinear problems [21,22,23]. The experimental results show that the digital-imaging-based color fastness grading method proposed in this paper has better performance when compared to the existing methods.

## 2. Methodology

**Figure 1:**
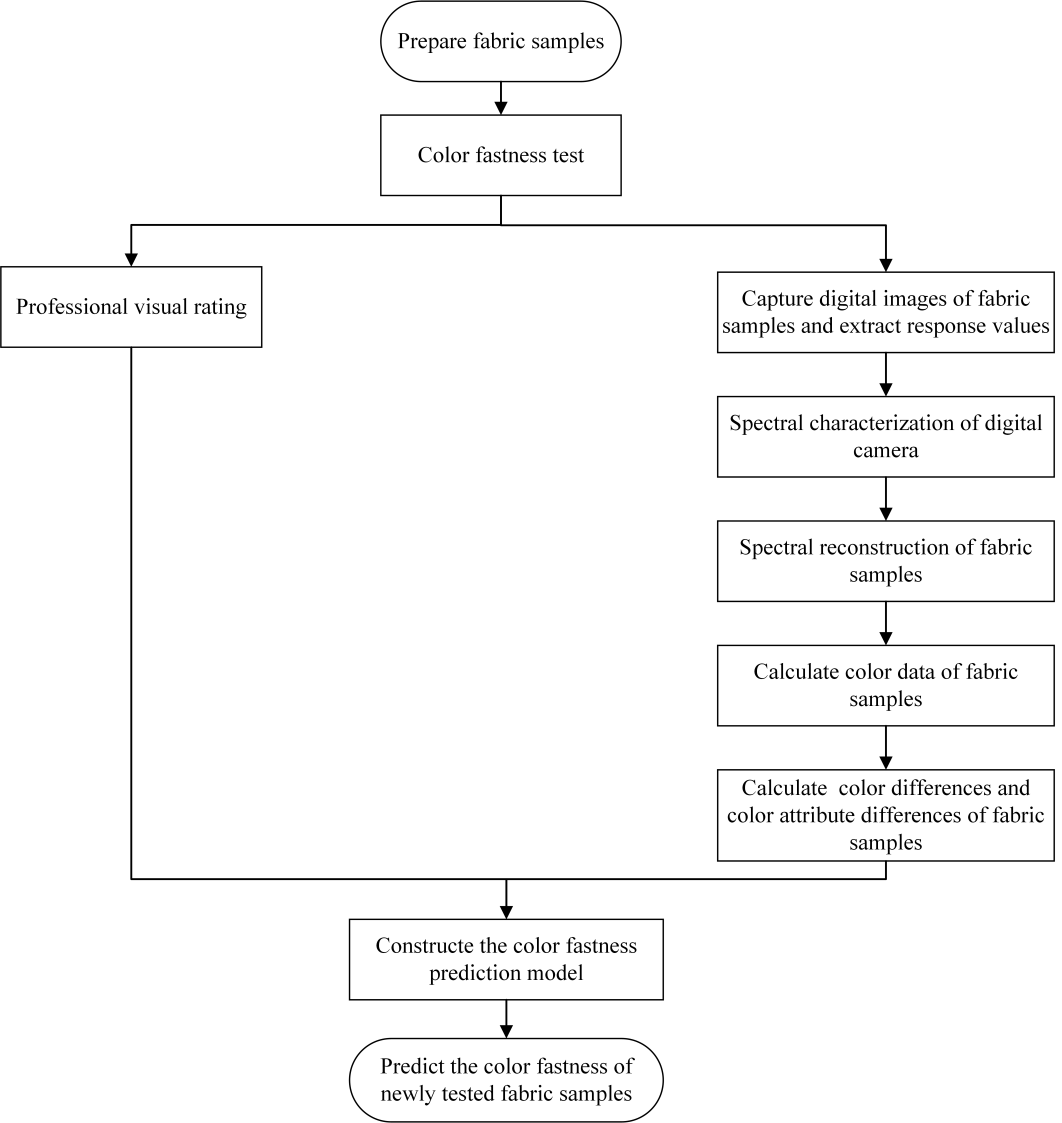
The overall flowchart of the proposed color fastness digital grading method.

Conventional color fastness mainly includes color fastness to rubbing, color fastness to washing, color fastness to perspiration, and color fastness to light [24,25,26], etc. This paper mainly studies the color fastness to rubbing. The overall flowchart of the proposed method is shown in Figure 1. Firstly, the fabric samples are prepared for color fastness modeling. The fabrics are paired samples; one of them will be rubbed as tested and another remains as a reference. After the rubbing test, all the samples will be visually graded by a specially trained professional under standard grading conditions. So, the ground truth of each tested sample is acquired as a reference for digital grading model construction. Then, the spectral reconstruction technology is used to reconstruct the spectral reflectance of the modeling samples, and the color data of the modeling sample are calculated by colorimetry theory. Based on the calculated color data, we can acquire the color difference (such as CIEDE2000) and color attribute difference (such as ΔL*, Δa*, and Δb*) of each paired sample. Finally, the BP neural network is adopted to construct the color fastness model between the input data (color difference and color attribute difference) and the visually graded color fastness result. Using the constructed color fastness prediction model, the color fastness grade of the newly tested samples will be easily predicted.

Details of color data acquisition based on spectral reconstruction technology are presented in Section 2.1, and the construction of the color fastness digital grading model based on the BP neural network is illustrated in Section 2.2. In addition, a brief introduction to the current two types of digital-imaging-based methods is also presented in Section 2.2.

### 2.1. Color Data Acquisition Based on Spectral Reconstruction

**Figure 2:**
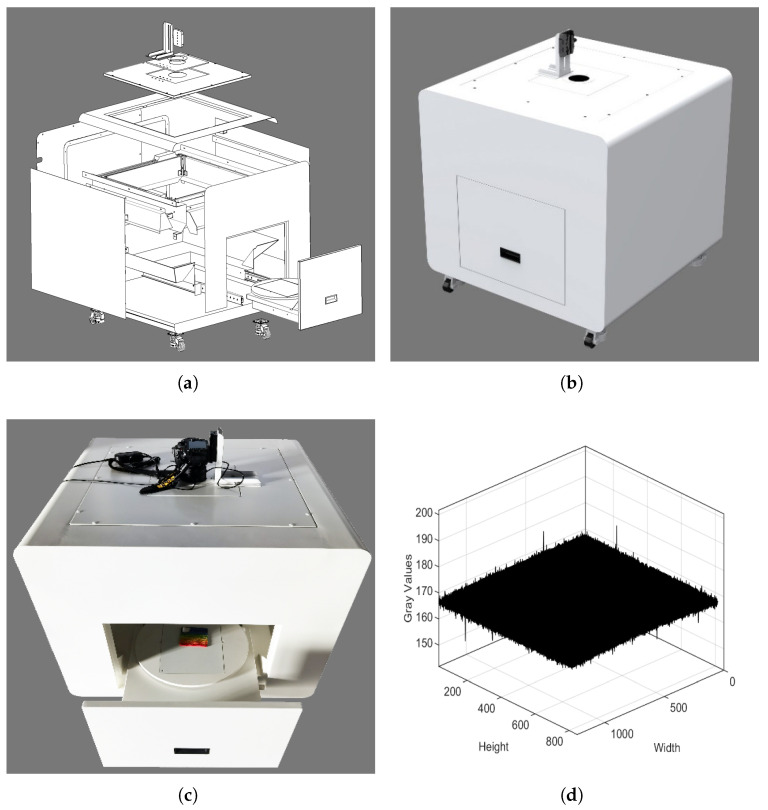
(**a**) Left up: the geometric diagram of the uniformly illuminated light box; (**b**) right up: the real rendering effect of light box; (**c**) left down: the real product of light box; and (**d**) right down: the inner illumination uniformity over the imaging area over the platform.

In this study, spectral reconstruction technology was used to calculate the color data of fabric samples. The first step is to take digital images of fabric samples and spectral characterization samples (such as the X-rite ColorChecker color chart or custom fabrics chart) with a digital camera. With uniform illumination of daylight light source in a closed light box of ColorEye, the sample is placed on the sample platform and the optical path of the digital camera is perpendicular to the plane of the platform. The geometric diagram of the uniformly illuminated light box is shown in Figure 2a, and the rendering effect of light box is shown in Figure 2b. The real product of light box is presented in Figure 2c and the inner illumination uniformity data of the imaging area over the platform checked with the X-rite gray card and Nikon D7200 digital camera are plotted in Figure 2d. To set the appropriate imaging parameters, such as ISO, shutter speed, and aperture size, we make sure the RGB values of the white and black patch in the X-rite ColorChecker 24 color chart are approximately 235 and 35, respectively. Therefore, we set the ISO as 100, the shutter speed as 1/20 s, and the aperture size as f/5.6 with a focal length of 35 mm. In the experiment, we use these imaging parameters to capture the samples.

Using the set imaging parameters, the digital images of fabric samples and spectral characterization samples are captured in the light box by a digital camera, and the average RGB values of each fabric sample and each color patch in the modeling samples are extracted. If we set the extract area as m × n pixels, the average RGB response values in the extraction area are calculated as shown in Equation (1) ,

(1) $$d=\frac{1}{m\times n}\sum_{i=1}^{m\times n}\left(r_{i},g_{i},b_{i}\right),\;$$

where *i* indicates the *i*th pixel in the extracted area, r${}_{i}$, g${}_{i}$, and b${}_{i}$ are the red, green, and blue channel RGB response values of the *i*th pixel, and **d** is the response value vector with the dimension of 1 × 3. It should be noted that the raw format digital response values without being post-processed by the digital camera ISP (Image Signal Processing) module are used in this study. Compared to the normal RGB response values commonly used in current methods, the raw format response values are cleaner and have more linearity than post-processed RGB values, which will benefit the higher spectral reconstruction accuracy. The response of each channel is no longer linear after processing the raw image but is better represented by a more complex non-linear law. Additionally, the post-processing methods of different camera manufacturers often differ and are difficult to accurately simulate or describe, making it challenging to model post-processing steps accurately [27,28].

In this study, the spectral characterization of a digital camera is carried out utilizing a traditional polynomial-based regularized pseudo-inverse technique [29]. As an illustration, the raw response value from the spectral characterization sample is extended into a second-order polynomial, as shown in Equation (2), which has a total of 10 expansion items.

(2) $$d_{\exp}={\left(1,r,g,b,rg,rb,gb,r^{2},g^{2},b^{2}\right)}^{T},\;$$

where **d**${}_{exp}$ is the vector of digital response values following polynomial expansion, the superscript *T* denotes the transpose, and *r*, *g*, and *b* are the red, green, and blue channel raw response values of any sample. Equation (3) is the expanded matrix of digital response values of the spectral characterization samples after polynomial expansion.

(3) $$D_{\mathrm{train}}={\left(d_{\exp,1},d_{\exp,2},\ldots ,d_{\exp,j}\right)}^{T}\left(j=1,2,\ldots ,P\right),$$

where *j* is the *j*th spectral characterization sample, P indicates the total number of spectral characterization samples, **d**${}_{exp,j}$ stands for the extended vector of numerical response values for the *j*th sample, and **D**${}_{train}$ is the extended matrix of spectral modeling samples.

As indicated in Equations (4)–(7), in the proposed method, we use the Tikhonov regularization to regularize the solution of the spectral reconstruction matrix. Firstly, the singular value decomposition (SVD) algorithm is applied to the expanded response matrix **D**${}_{train}$ of the spectral modeling samples. Then, a very small number α is added to the eigenvalues to obtain the constrained eigenvalues to reduce the condition number of the expanded response matrix. After that, we reconstruct the response expansion matrix **D**${}_{train,rec}$. Finally, the spectral reconstruction matrix **Q** is obtained by solving with pseudo-inverse (PI) algorithm and to obtain the spectral reconstruction model.

(4) $$D_{\mathrm{train}}={\mathrm{USV}}^{T},$$

(5) P=S+αI,

(6) $$D_{\mathrm{train},\mathrm{rec}\;}=\mathrm{UPV}{}^{T},\;$$

(7) $$Q=R_{\mathrm{train}\;}\times \mathrm{pinv}\left(D_{\mathrm{train},\mathrm{rec}\;}\right),$$

where **R**${}_{train}$ is the spectral matrix of the spectral modeling samples, **U** and **V** are the orthogonal decomposition matrices obtained by SVD algorithm, **S** and **P** are diagonal matrices containing eigenvalues, **I** is the unit matrix, and *pinv*(·) is the mathematical function of pseudo-inverse algorithm.

In the next step, we use the established spectral reconstruction model to reconstruct the spectral reflectance of the newly tested fabric sample. The extracted raw response **d**${}_{test}$ of the newly tested fabric is first expanded using the polynomial as in Equation (1) to obtain the expanded response vector **d**${}_{test,exp}$. Then, the spectral reflectance of tested fabric samples is reconstructed using the spectral reconstruction matrix **Q**, as indicated in Equation (8).

(8) $$r_{\mathrm{test}\;}=Q\times d_{\mathrm{test},\exp\;},$$

where **r**${}_{test}$ is the reconstructed spectral reflectance of the tested fabric sample and matrix **Q** is the established spectral reconstruction matrix.

Using the constructed spectral reflectance above, the corresponding color data of the fabric samples are calculated based on the colorimetry theory. The tristimulus values of the fabric samples are calculated as indicated in Equations (9) and (10).

(9) $$\begin{array}{cc} \; & X=k\int_{\lambda }x\left(\lambda \right)E\left(\lambda \right)S\left(\lambda \right)d\lambda \\ \; & Y=k\int_{\lambda }y\left(\lambda \right)E\left(\lambda \right)S\left(\lambda \right)d\lambda \\ \; & Z=k\int_{\lambda }z\left(\lambda \right)E\left(\lambda \right)S\left(\lambda \right)d\lambda \end{array},$$

where

(10) $$k=100/\left[\sum_{\lambda }y\left(\lambda \right)E\left(\lambda \right)d\lambda \right],$$

where *x(λ)*, *y(λ)*, and *z(λ)* are standard observer color matching functions, *E(λ)* is the fabric samples spectral reflectance, *S(λ)* is the relative spectral power distribution function of the light source, λ is the wavelength, *k* is the adjustment factor, and *X*, *Y*, and *Z* are the three tristimulus value data of the fabric sample.

Then, the CIELab color data of the fabric samples are calculated from the corresponding tristimulus. According to the theory of chromaticity, the method of calculating the corresponding CIELab color data from the tristimulus value data is shown in Formula (11) to Formula (12).

(11) $$\begin{array}{cc} \; & L^{*}=116f\left(\frac{Y}{Y_{n}}\right)-16\\ \; & a^{*}=500\left[f\left(\frac{X}{X_{n}}\right)-f\left(\frac{Y}{Y_{n}}\right)\right],\\ \; & b^{*}=200\left[f\left(\frac{Y}{Y_{n}}\right)-f\left(\frac{Z}{Z_{n}}\right)\right] \end{array}$$

where

(12) $$\left\{\begin{array}{ccc} f\left(\frac{H}{H_{n}}\right)={\left(\frac{H}{H_{n}}\right)}^{1/3} & \;\mathrm{if}\; & \left(\frac{H}{H_{n}}\right)>{\left(24/116\right)}^{1/3}\\ f\left(\frac{H}{H_{n}}\right)=\left(841/108\right)\left(\frac{H}{H_{n}}\right)+16/116 & \;\mathrm{if}\; & \left(\frac{H}{H_{n}}\right)\leq {\left(24/116\right)}^{1/3} \end{array},\right.$$

where L*, a*, and b* represent the lightness, red–green, and yellow–blue color values of the fabric sample in the CIELab color space, respectively. *X*, *Y*, and *Z* are the three stimulus value data of the fabric, and *X${}_{n}$*, *Y${}_{n}$*, and *Z${}_{n}$* are the three stimulus value data of the reference light source. In Equation (12), *H* and *H${}_{n}$* represent the three stimulus values of the fabric and reference light source, respectively.

With the CIELab color data of fabric samples, the next step is to calculate the color difference and color attribute difference in order to perform the color fastness test. Using the CIELab color data of the fabric sample pair, the CIEDE2000 color difference value of the fabric sample pair and the corresponding ΔL*, Δa*, and Δb* color difference values are calculated. The CIEDE2000 color difference formula is shown in formula (13).

(13) $$\Delta E_{2000}^{*}={\left[{\left(\frac{\Delta L^{\prime }}{k_{L}S_{L}}\right)}^{2}+{\left(\frac{\Delta C^{\prime }}{k_{C}S_{C}}\right)}^{2}+{\left(\frac{\Delta H^{\prime }}{k_{H}S_{H}}\right)}^{2}+R_{T}\left(\frac{\Delta C^{\prime }}{k_{C}S_{C}}\right)\left(\frac{\Delta H^{\prime }}{k_{H}S_{H}}\right)\right]}^{\frac{1}{2}},$$

where Δ*L${}^{\prime }$*, Δ*C${}^{\prime }$*, and Δ*H${}^{\prime }$* represent the lightness, chroma, and hue differences in the fabric sample pair in the CIELCh color space, which are automatically converted from the CIELab color space when calculating the color difference. *k${}_{H}$*, *k${}_{L}$*, and *k${}_{C}$* are the weights for hue, lightness, and chroma when calculating the color difference. For fabric samples, *k${}_{L}$* is usually set to 1.5, and *k${}_{H}$* and *k${}_{C}$* are set to 1. *S${}_{L}$*, *S${}_{C}$*, and *S${}_{H}$* are the weighting functions for lightness, chroma, and hue, respectively, and *R${}_{T}$* is the adjustment term for color difference calculation. The calculations of the color differences ΔL*, Δa*, and Δb* are shown in Equations (14)–(16).

(14) $$\Delta L^{*}=L_{1}^{*}-L_{2}^{*},\;$$

(15) $$\Delta a^{*}=a_{1}^{*}-a_{2}^{*},$$

(16) $$\Delta b^{*}=b_{1}^{*}-b_{2}^{*},\;$$

where (L*${}_{1}$, a*${}_{1}$, b*${}_{1}$) and (L*${}_{2}$, a*${}_{2}$, b*${}_{2}$) are the CIELab color data of the reference sample and the color fastness test sample in the fabric sample pair, respectively.

### 2.2. Color Fastness Prediction Methods

#### 2.2.1. Existing Methods

- The color difference conversion method.

For the color difference conversion method, the RGB values of the target sample images are first extracted as described in Equation (1). Then, the RGB values are converted to CIEXYZ stimulus values according to the conversion method between the working RGB color space and CIEXYZ color space. However, since the CIEXYZ is not a perceptually uniform color space, we therefore further transform the CIEXYZ to the device-independent and perceptually more uniform CIELab color space; this will benefit to calculate the color difference in accordance with the visual perception. After we have the CIELab color data, the color difference in the paired sample is calculated using the CIE DE2000 color difference equations. Then, the color fastness grade of staining is calculated according to the relevant regulations in ISO 105-A11 standard [30], and the final rating result is obtained [10,11]. The specific algorithm formula is shown in Equations (17) and (18):

(17) $$GRS=-0.061\Delta E_{GRS}+2.474\left(1+e^{-0.191\Delta E_{GRS}}\right),$$

where

(18) $$\Delta E_{GRS}=\Delta E_{00}-0.423\sqrt{\Delta E_{00}^{2}-\Delta L_{00}^{2}},$$

where *GRS* stands for calculated grade, Δ*E${}_{00}$* is the color difference value calculated using the CIEDE2000 color difference formula.

- The gray scale difference method.

The gray scale difference method is proposed based on the concept that the color fastness grade is highly correlated to the gray scale difference in the standard grading gray card [12,13], where the further assumption is that the gray scale difference in the standard grading gray card is highly correlated with the color difference. Based on the above assumptions, one should first capture the image of standard grading gray card and calculate the gray scale difference in the paired gray patches for each color fastness grade. Then, the polynomial-based gray scale difference method is applied to the corresponding gray scale difference and the color fastness grade to construct the color fastness prediction model. With the constructed model, the color fastness grade of the testing paired sample will be predicted through calculating the gray image difference and putting it into the prediction model. It should be noted that the imaging of the paired testing sample should be captured at the same imaging conditions as the prediction model is constructed or the prediction result will be incorrect.

In addition, except for the gray scale difference method itself, we also use the ΔL* to replace the gray scale difference to establish the fitting model between the visual grade rating results and the ΔL* of paired staining samples. This is to test whether we can directly construct the prediction model from ΔL* of paired staining samples to the visual grade rating results.

#### 2.2.2. The Proposed Method

In this study, the BP neural network is used to construct a color fastness prediction model for fabric samples. The BP neural network is a multi-layer feedforward network trained using the backpropagation algorithm. It typically consists of three layers, namely the input layer, hidden layer, and output layer. Each layer of neurons is fully connected to the adjacent layer, but there is no connection among neurons in the same layer. The neurons in different layers are not connected in a feedback manner, forming a hierarchical and feedforward neural network system [21,31].

The BP neural network is a useful tool for both classification and regression problems. In the case of color fastness rating, it can be approached as either a classification or regression problem. When we treat the color fastness rating as classification problem, of which the color fastness grade is divided into nine grades ranging from 1 to 5 with 0.5 as the step, we can obtain the color fastness grade of test samples directly, but it is not beneficial for us to evaluate the precision of the prediction model more accurately. Therefore, we treat the color fastness rating as a regression problem in this study, and the prediction model is trained by the BP neural network and prepared training samples. The specific predicted color fastness values of the testing paired samples between 0 and 5 will be provided by the prediction model with continuous numbers. Further, based on the predicted color fastness value, the color fastness grade will be determined to the nearest grade level.

### 2.3. Evaluation Metrics

In this study, the general metric of root mean square error (RMSE) is used to measure the deviation between the predicted color fastness grade and the visual grade of fabric samples obtained from the experts. The calculation of RMSE is shown in Equation (19):

(19) $$RMSE=\sqrt{\frac{\sum_{i=1}^{n}{\left(y-y_{1}\right)}^{2}}{n}},$$

where *RMSE* represents the root mean square error, *y* represents the predicted color fastness value of the tested samples, *y${}_{1}$* represents the actual value of the tested samples, and *n* represents the number of tested samples. The smaller the *RMSE* value, the better the consistency between the model prediction results and the visual rating results. In addition, the absolute value of the predicted error of each tested fabric is also calculated when we compare each method statistically.

## 3. Experiment

To validate the proposed fabric color fastness rating method for rubbing tests, we employed the following instruments and materials in the experiment. They are the Y(B) 571-III color fastness rubbing tester, the pure cotton twill fabric with the size of 10 × 25 cm, the white cotton rubbing cloth with the size of 5 × 5 cm , the standard gray card for assessing staining of color, the standard colorimetric light box ColorChex N7, the Nikon D7200 digital camera, the X-rite Color Checker classical 24 color chart, and the closed lighting box ColorEye with uniform illumination. Using the above instruments and materials, the experiment aimed to assess the color fastness of the textile fabric after multiple rubbing tests and verify the accuracy of the proposed rating method. The rubbing fastness experiment is carried out in Section 3.1, the visual rating experiment is described in Section 3.2, the construction of prediction model based on BP neural network is illustrated in Section 3.3, and the testing of the existing methods is presented in Section 3.4.

### 3.1. The Rubbing Color Fastness Experiment

**Figure 3:**
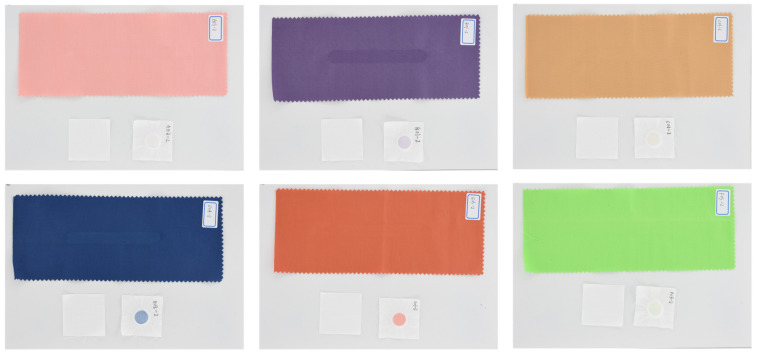
Digital images of the rubbed samples in the database; for each sample, the rubbed samples and their corresponding color-stained rubbing cloth are presented.

The color fastness to rubbing is an important testing item for fabric. It is the degree of color retention of fabrics after they are subjected to rubbing during use. Usually, the color fastness to rubbing of fabrics is reflected by the staining grade. According to the ISO 105-X12 standard [32], dry rubbing and wet rubbing tests should be tested simultaneously [22,23]. The testing parameters of the fabric samples are shown in Table 1. During the testing process, the tested fabric samples are fixed to the rubbing platform of the color fastness rubbing tester of Y (B) 571-III, with a dry rubbing cloth on the one rubbing head and a wet rubbing cloth with a water content of 95%–100% on the other rubbing head. Through adjusting the number of rubbing times between 150 and 200 with the pressure of 9 N, we can obtain the experiment samples with the color fastness grades covering all nine steps from 1 to 5 with the 0.5 step. Finally, the database including the rubbed samples and their corresponding color-stained rubbing cloth were constructed; some of the samples are shown in Figure 3.

### 3.2. The Visual Rating Experiment

**Figure 4:**
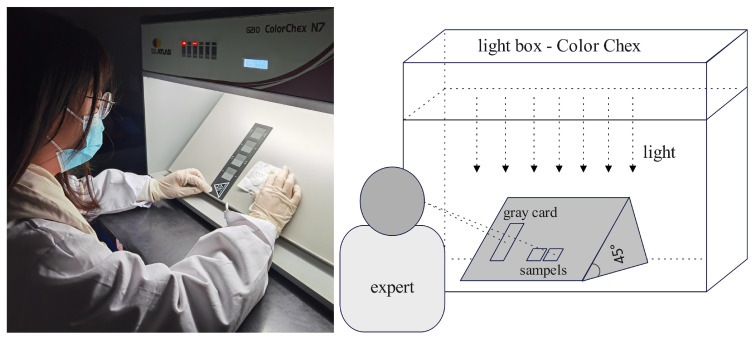
The scene and geometric diagram of visual rating experiment settings.

According to standard ISO 105-A03 [33], the gray card for staining is used to visually evaluate the rubbered cloth samples. The visual rating experiment is carried out in a dark room and in a professional light box ColorChex N7 (see as in Figure 4). Using D65 standard light source, the professional rating person sits in front of the lighting box to visually rate the color fastness grade of the sample. The vertical viewing distance from the eyes to the viewing surface is about 30 cm. During the rating, the unstained lining fabric and the stained lining fabric were placed side by side in the same plane, while the gray sample card was also placed nearby on the same plane. The color difference between the unstained lining fabric and the stained lining fabric was visually assessed according to the difference level on the standard grading gray card, and the reference color fastness grade was acquired of all the modeling and testing fabric samples through the visual rating experiment.

### 3.3. The BP Neural Network Modeling

#### 3.3.1. Data Preprocessing

We produced a total of 70 groups of rubbing samples, and the sample diagram is shown in Figure 3. We used the colorimetry theory introduced previously to calculate the relevant color data values of the sample image. Then, we performed simple preprocessing on the data, that is, normalization of the CIEDE2000, ΔL*, Δa*, and Δb*. We adopted the min–max normalization method, as shown in Equation (20):

(20) $$x^{\prime }=\left(x-\min\right)/\left(\max-\min\right),$$

where *x${}^{\prime }$* is the normalized value, *x* is the original value, min is the minimum value of all the input values, and max is the maximum value of all the input values. The normalized input values of CIEDE2000, ΔL*, Δa*, and Δb* will be scaled to the range [0–1].

#### 3.3.2. Model Building and Training

**Figure 5:**
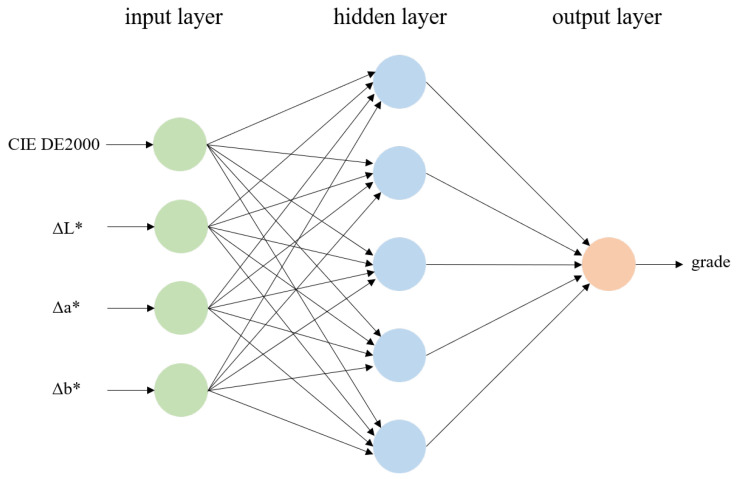
BP neural network structure diagram.

In this study, the CIEDE2000 color difference value and the ΔL*, Δa*, and Δb* values of fabric samples are used as input, and the corresponding visual rating grades of fabric samples were used as output to train the BP neural network. According to the number of input and output parameters, we set the number of input layer nodes to 4, and the number of output layer nodes to 1. The number of hidden layer nodes is set to 5. The structure diagram of the training model is shown in Figure 5.

During the training process, we repeatedly adjust the number of iterations to balance the model’s generalization ability and training effect. Through experiments, we finally set the maximum number of iterations to 1000. The learning rate is set to 0.001, and the Sigmoid function is selected as the activation function in the hidden layer neurons; the Sigmoid function is shown in Equation (21).

(21) $$S\left(x\right)=\frac{1}{1+e^{-x}},$$

where *S(·)*is the Sigmoid activation function, *x* is the independent variable, and *e* is the natural logarithm. The weights between the nodes of the BP neural network were continuously adjusted based on the root mean square error of the modeling samples until the overall average error of the modeling samples reached a stable convergence state. When the BP neural network reaches the convergence state, the color fastness prediction model is constructed.

### 3.4. Testing of Existing Methods

#### 3.4.1. Testing of Color Difference Conversion Method

**Figure 6:**
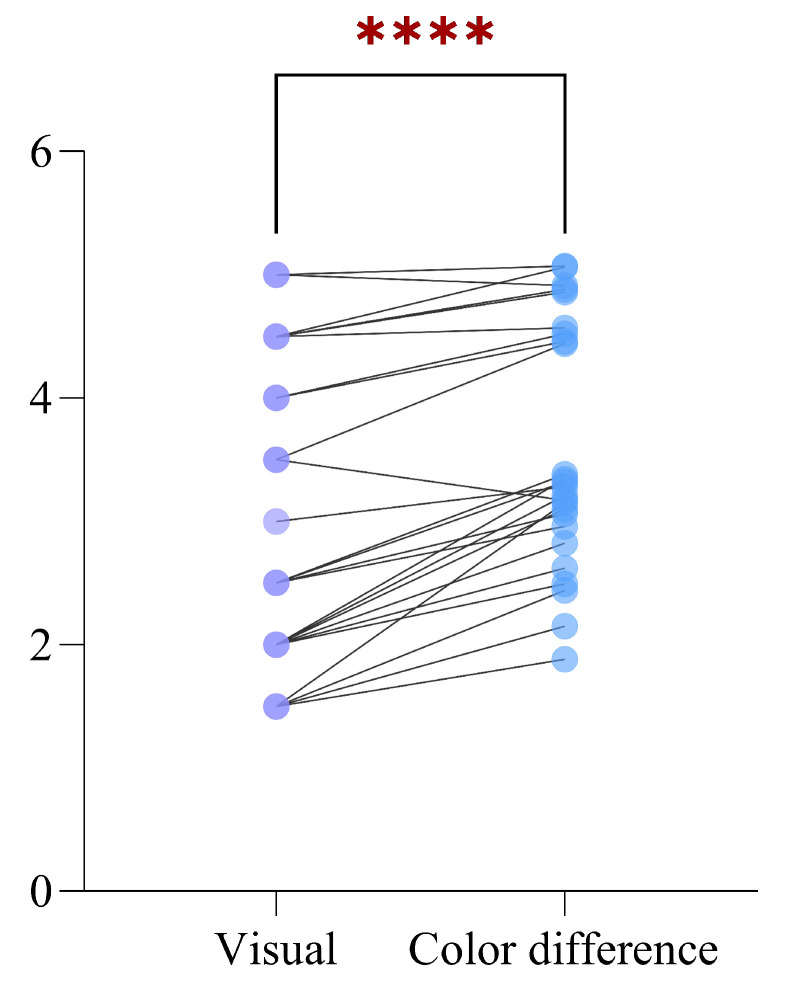
Results of the paired-sample *t*-test between prediction results of color difference conversion method and visual rating results using 25 randomly selected fabric samples.

The color data of all the fabric samples were extracted using the method described in Section 2.1, and the color fastness prediction results were acquired using the color difference conversion method described in Section 2.2.1. To validate the performance of the color difference conversion method, a paired-sample *t*-test was conducted between the prediction results and the visual results. Firstly, 25 samples are randomly selected from all fabric samples, and they satisfy the normal distribution condition after normal distribution test. Then, the paired-sample *t*-test was performed to compare whether the prediction results and visual results were significantly different from each other, and the result of paired-sample *t*-test is shown in Figure 6.

In Figure 6, the purple dot on the left represents the color fastness of the visual rating, the blue dot on the right represents the predicted color fastness of the color difference conversion method, and each line between them connects the same sample. The ‘****’ above the straight line indicates that the *p*-value of the paired sample *t*-test is less than 0.0001, which means that there is a significant difference between the prediction results of color difference conversion method and visual rating results. Therefore, a further step is needed to modify the prediction results of color difference conversion method to the visual rating results.

The color difference conversion method is proposed based on the calculation of the CIEDE2000 color difference from the color attributes of L*, a*, and b* in the CIELAB color space. To realize the modification from the prediction results of color difference conversion method to the visual rating results, the ΔL*, Δa*, and Δb* were selected to modify the prediction results of color difference conversion method. The modification of the predicted result is based on a linear regression model, where the ΔL*, Δa*, and Δb* are treated as the independent variables, and the grade difference in color fastness between predicted and visual rated is treated as the dependent variable. The results of linear regression analysis between independent and dependent variables are presented in Table 2.

It can be seen from Table 2 that the *p*-value of the significance analysis of ΔL* is less than 0.05, indicating a significant impact on the grade difference in color fastness between predicted and visual rated results. The *p*-values of Δa* and Δb* are greater than 0.05, and the regression coefficient is not significant, indicating that the Δa* and Δb* have little influence on the grade difference in color fastness. At the same time, the VIFs are all less than 5, indicating that there is no multicollinearity among the independent variables. According to the linear regression analysis results, the multiple linear regression model is constructed as shown in Equation (22).

(22) $$Y=-0.121-0.034X_{1},$$

where $X{}_{1}$ represents the ΔL*, and *Y* represents the grade difference in color fastness between predicted and visual rated results. Therefore, with the established linear regression model, the predicted color fastness based on color difference conversion method can be easily modified to the visual rating grade. The five-fold cross-validation is also performed for the color difference conversion method when compared with the proposed method in Section 4.

#### 3.4.2. Testing of Gray Scale Difference Method

**Figure 7:**
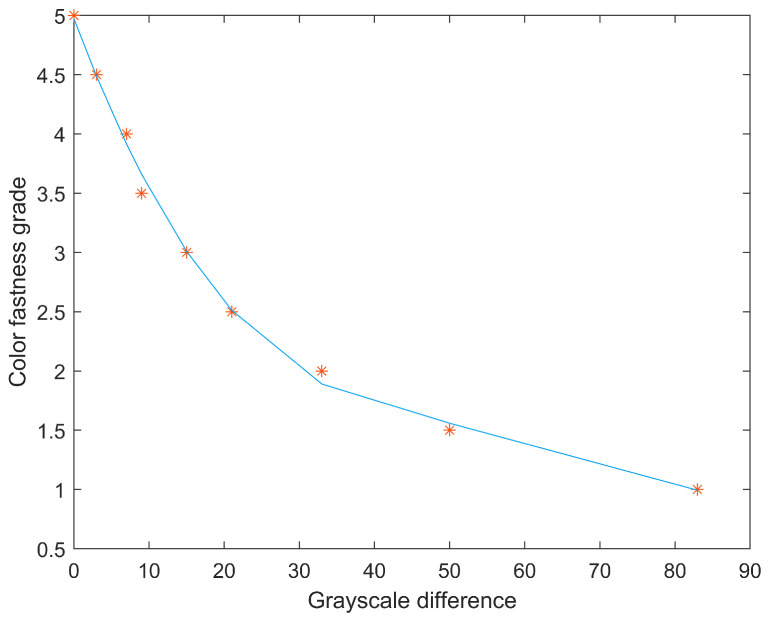
The third-order polynomial curve to fit the relationship between gray scale difference in standard grading gray card and the corresponding color fastness grade.

For testing of the gray scale difference method, the standard grading gray card images were first collected and converted into gray scale images. The gray scale difference between two patches of each grade was extracted, and the gray scale difference was fitted to the corresponding color fastness grade using a curve fitting method. According to the tests, the third-order polynomial curve was found to have the best fitting effect, as shown in Figure 7. The red dots in the figure represent the grayscale difference between the two pieces at each grade of the gray card.

The analysis of the third-order polynomial fitting is presented in Table 3. Based on the fitted parameters, a third-order polynomial fitting equation was obtained, where *x* represents the gray scale difference in the fabric sample to be evaluated, *p*1, *p*2, *p*3, *p*4 are the coefficients of the equation, and the predicted color fastness grade is denoted as *D*. The correlation coefficient of the fitting equation of $R{}^{2}$ is 0.99, indicating a good fitting result between the gray scale difference and the color fastness grade. The next step is to convert the testing fabric sample into a gray scale image, and to extract its gray scale value. After that, the color fastness grade of the testing fabric sample will be predicted using the established third-polynomial prediction model.

Furthermore, in order to test whether we can directly construct the prediction model from the ΔL* of paired staining samples to the visual grade rating results, we also used the ΔL* of the paired staining samples to replace the gray scale difference in them to fit with the visual grade rating results based on polynomial regression.

## 4. Results and Discussion

**Figure 8:**
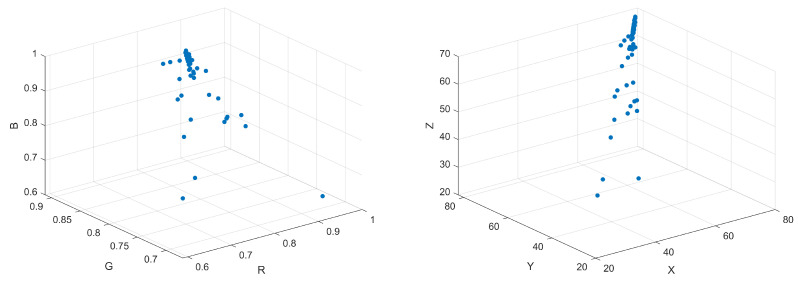
The color distribution of 70 samples in RGB space (**left**) and CIEXYZ color space (**right**).

In this study, a total of 70 groups of samples were produced to study the staining fastness grade of rubbing. The color data values of the sample images were extracted using the principle of spectral reconstruction, and then a prediction model was built through the BP neural network. The RGB and XYZ space color values of 70 groups of samples are shown in Figure 8.

**Figure 9:**
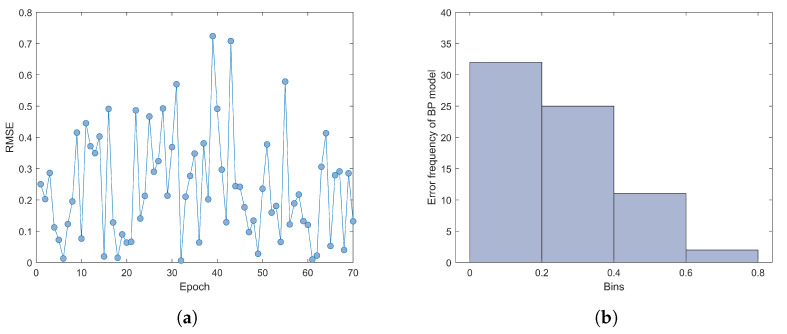
(**a**) The predicted error distribution of the proposed method tested with five-fold cross-validation; (**b**) predicted error histogram of proposed method.

Due to the limited number of samples, we used five-fold cross-validation to obtain the predicted values of 70 groups of samples. Five-fold cross-validation divides the dataset into five subsets, four of which are used to train the model, while the remaining one is used to evaluate the performance of the model. After training, the average root mean square error of the final five training results is 0.29. We plotted the error scatterplot of 70 groups of samples as shown in Figure 9a. At the same time, to understand the error distribution more clearly, we drew an error histogram as shown in Figure 9b. A histogram uses a series of rectangles with equal width and different heights to represent data. The width represents the group interval, and the height represents the number of samples within the specified group interval.

Figure 9a shows that most of the sample prediction errors are distributed below 0.5, and the prediction error of a few samples exceeds 0.5. From Figure 9b, it can be further seen that there are more than 30 groups of sample error predictions between 0 and 0.2. Among them, the prediction error of twenty-five groups of samples is in the range of 0.2 to 0.4, eleven samples of the prediction error are between 0.4 and 0.6, and only two samples of the prediction error are located between 0.6 and 0.8.

**Figure 10:**
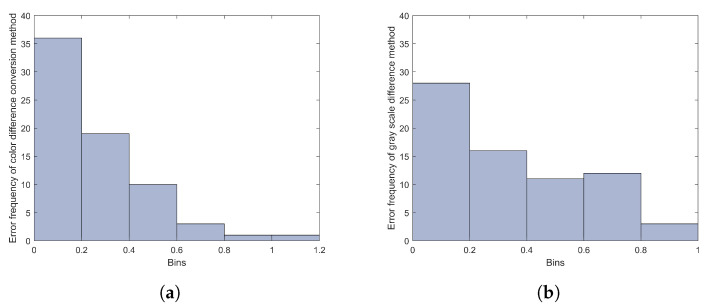
(**a**) The predicted error histogram of color difference conversion method; (**b**) the predicted error histogram of gray scale difference method.

We also calculated the predicted errors of 70 groups of samples. The global version of the color difference conversion method and the gray scale difference method itself as introduced in Section 2.2.1 and the histogram of their prediction errors are plotted in Figure 10.

Figure 10a,b represent the sample error histograms of the color difference conversion method and the gray scale difference method, respectively. It can be seen that, for the color difference conversion method, even though there are more than 35 groups of samples with a predicted error smaller than 0.2, there are still five samples with a predicted error larger than 0.6, and two of them even larger than 0.8. It seems that the error distributions of the color difference conversion method are more dispersed than the proposed method. For the gray scale method, it looks like the same trend, and the prediction result of the gray scale method is much worse as the average of the RMSE of it reaches 0.42, which is much larger than the proposed method of 0.29 and the color difference conversion method of 0.34.

**Figure 11:**
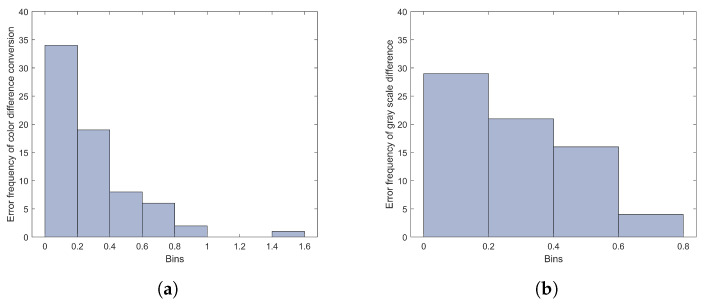
(**a**) The predicted error histogram of color difference conversion method tested with five-fold cross-validation; (**b**) the predicted error histogram of the optimized gray scale method tested with five-fold cross-validation.

To more fairly compare with the proposed method, five-fold cross-validation is performed for both the color conversion method and the optimized gray scale method, where the gray scale difference is replaced with ΔL* of the paired staining samples. For the color difference conversion method, the first step is to calculate the GRS using Equations (17) and (18) based on ISO 105-A11 [30], and then the calculated GRS is mapped to the visual rating results based on regression. We use the five-fold cross-validation method to test the regression method, and, for each fold during the test, the regression model is constructed using 56 training samples and the remaining 14 samples are used as tests. This is the same validation strategy for the optimized gray scale difference method, where, during the five-fold cross-validation, the third-order polynomial is used to fit the model using 56 samples and the remaining 14 samples are used as testing. After all the five-fold cross-validation, we can calculate the prediction errors of all the samples. The prediction error histograms of the two methods are shown in Figure 11.

As can be seen from Figure 11a, for the color difference conversion method after five-fold cross-validation, there are 34 sample error values below 0.2, 19 sample error values between 0.2 and 0.4, and a few sample error values exceeding 0.4. At the same time, there is one sample whose error value is relatively large, between 1.4 and 1.6. For the optimized gray scale difference method in Figure 11b, 29 groups of samples of predicted error are less than 0.2, 20 groups of samples are between 0.2 and 0.4, and the remaining 21 groups sample between 0.4 and 0.8.

**Figure 12:**
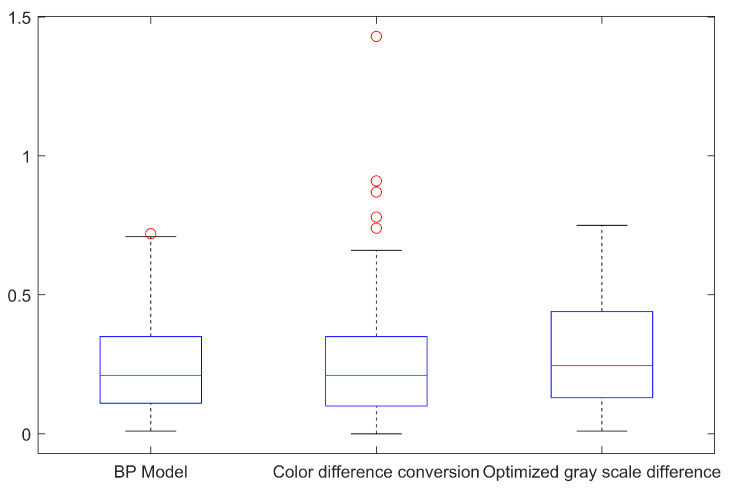
Boxplot of predicted error of proposed BP model, color difference conversion, and optimized gray scale difference method tested with five-fold cross-validation.

In order to better compare the results of the five-fold cross-validation of the three methods, we provide the boxplot of the predicted error of the three methods in Figure 12. Boxplots are a stable method of describing the distribution of data and are not affected by outliers. For a boxplot, the upper boundary of the box represents the upper quartile of the data, while the lower boundary of the box represents the lower quartile of the data. The line in the middle of the box represents the median value of the data. The upper and lower limits of the box represent the maximum and minimum values of the data. The points outside the box (represented by filled circles in Figure 12) can be considered as outliers, which are typically defined as values that fall below the lower quartile minus 1.5 times the interquartile range or above the upper quartile plus 1.5 times the interquartile range.

As can be seen from Figure 12, the predicted error distributions of the BP neural network and the color difference conversion method are relatively concentrated, while the predicted error distribution of the optimized gray scale difference method is relatively dispersed. At the same time, the average predicted error of the BP model and the color difference conversion method is lower, while the average predicted error of the optimized gray scale difference method is higher. Comparing the BP model and the color difference conversion method, we can see that the color difference conversion method has more outlier points, that is, points with larger errors.

To further compare the five methods tested in this study, we have also summarized all the statistical results of the predicted error of each method in Table 4. They are the average prediction error, maximum error, minimum error, 90 percent error (referring to the predicted error of the 63rd sample after arranging the 70 sample errors sorted from minimum to maximum), and standard deviation. As can be seen from the table, the average error, maximum error, and standard deviation of the BP model are all the smallest. The average error of the gray scale difference method is the largest, which is 0.34. The maximum error of the color difference conversion with five-fold cross-validation is the largest, which is 1.43. The minimum error of all five tested methods is equal to zero or very near to zero, and, for the 90% error, the BP method and the optimized gray scale difference method are almost equal to each other, and obviously smaller than the other three methods. However, upon analysis together with the boxplot of the predicted error in Figure 12 and the predicted error histogram, we can see that the BP model is still better than the optimized gray scale difference method.

Through the fairly comparative analysis of the different methods above, it is concluded that the color fastness prediction method based on spectral reconstruction technology and BP neural network has better performance than the existing method and the optimized version of them, which verifies that the predicted color fastness grades are consistent with the professional visual rating result. It also indicates that the color fastness rating method based on spectral reconstruction and BP neural network can be a good choice in the future to help with digital grading the color fastness regarding rubbing of fabrics.

## 5. Conclusions

In this study, the color fastness grading method was investigated and a new grading method based on spectral reconstruction technology and BP neural network modeling was proposed for digital grading the color fastness to rubbing of fabrics. The experimental results indicated the effectiveness and superiority of the proposed method. This method eliminates the subjective differences in traditional visual color fastness rating methods. Compared with the spectrophotometer method, the imaging-based color fastness evaluation method is more efficient and flexible. At the same time, the imaging-based color measurement method is also easily combined with imaging process technology to implement the other applications. Therefore, the research results of this article further enhance the feasibility of color fastness grading methods based on digital imaging in practical applications.

However, this study still has some shortcomings. Firstly, colorfastness to rubbing encompasses assessing change in color and staining. This study only discussed the staining color fastness to rubbing but did not study the color fastness to color change. Therefore, it has certain limitations. Secondly, traditional color fastness includes color fastness to rubbing, color fastness to light, color fastness to washing, etc. In this study, only color fastness to rubbing was discussed, and there was no relatively comprehensive study on color fastness. This is also what we need to consider in our next work. Finally, the number of samples used in this study is limited, which may affect the experimental results to a certain extent. Therefore, in future work, we will further increase the number of experimental samples to improve the experiment.

## Figures and Tables

**Table 1:** Parameters to test the fabric samples.

Texture	100% cotton twill
Size	10 × 25 cm
Yarn count	40 counts
Density	133 × 72
Color	pink, purple, yellow, blue, orange, green

**Table 2:** The results of linear regression analysis between independent and dependent variables.

Model	Unstandardized Coefficients		Standardized Coefficient	*t*	Significance (*p*-Value)	Covariance Statistics	
	B	Standard Error	β			Tolerance	VIF
Constant	−0.121	0.044		−2.777	0.007		
ΔL*	−0.034	0.009	−0.469	−3.779	0.000	0.637	1.57
Δa*	−0.021	0.017	−0.209	−1.234	0.222	0.343	2.92
Δb*	−0.014	0.014	−0.171	−0.985	0.328	0.325	3.075

**Table 3:** The analysis of the third-order polynomial fitting results.

Gray Scale Difference	Fitting Equation	Correlation Coefficient
third-order polynomial	$D=p1x^{3}+p2x^{2}+p3x+p4$ p1=−1.72e−05 p2=0.0029 p3=−0.17 p4=4.97	$R^{2}=0.99$

**Table 4:** Statistics of the predicted error of all the tested methods in this study.

	BP Model	Color Difference Conversion	Gray Scale Difference	Color Difference Conversion (Five-Fold)	Optimized Gray Scale Difference
Ave.	**0.24**	0.26	0.34	0.29	0.28
Max.	**0.72**	1.17	0.95	1.43	0.75
Min.	0.01	**0.00**	0.01	**0.00**	0.01
90%	0.49	0.54	0.71	0.63	**0.48**
Std.	**0.17**	0.22	0.26	0.26	0.18

Note: The bold vlues represent the best result for each statistic metric.

## Data Availability

The data used to support the findings of this study are available from the corresponding author upon request.
